# Cow-Pox

**Published:** 1840-11

**Authors:** 


### COW-POX.

It is melancholy to observe the apathy which prevails among the people and the profession, on the subject of vaccination. Such neglect deserves the severest rebuke. Thousands are growing up in the West, without being protected against small pox, although protection is so entirely within the reach of every individual from the cabin to the mansion. Verily, we are negligent people, in every thing but trade and politics. When the presidential election is over, it is to be hoped, we shall find time to get our children vaccinated.

